# The identification and characterization of a novel protein, c19orf10, in the synovium

**DOI:** 10.1186/ar2145

**Published:** 2007-03-15

**Authors:** Tracey Weiler, Qiujiang Du, Oleg Krokhin, Werner Ens, Ken Standing, Hani El-Gabalawy, John A Wilkins

**Affiliations:** 1 Department of Internal Medicine and Manitoba Centre for Proteomics and Systems Biology, University of Manitoba, Room 799, John Buhler Research Centre, 715 McDermot Avenue, Winnipeg, Manitoba, R3E 3P4, Canada; 2 Department of Physics and Astronomy, University of Manitoba, 510 Allen Building, Winnipeg, Manitoba, R3T 2N2, Canada; 3 Section of Rheumatology, Department of Internal Medicine, Faculty of Medicine, University of Manitoba, RR149 Rehab Hospital, 800 Sherbrook Street, Winnipeg, Manitoba, R3A 1M4, Canada

## Abstract

Joint inflammation and destruction have been linked to the deregulation of the highly synthetic fibroblast-like synoviocytes (FLSs), and much of our current understanding of the mechanisms that underlie synovitis has been collected from studies of FLSs. During a proteomic analysis of FLS cells, we identified a novel protein, c19orf10 (chromosome 19 open reading frame 10), that was produced in significant amounts by these cells. The present study provides a partial characterization of c19orf10 in FLSs, synovial fluid, and the synovium. Murine monoclonal and chicken polyclonal antibodies were produced against recombinant human c19orf10 protein and used to examine the distribution of c19orf10 in cultured FLSs and in synovial tissue sections from patients with rheumatoid arthritis or osteoarthritis. The intracellular staining pattern of c19orf10 is consistent with localization in the endoplasmic reticulum/Golgi distribution. Sections of rheumatoid arthritis and osteoarthritis synovia expressed similar patterns of c19orf10 distribution with perivascular and synovial lining staining. Double-staining *in situ *analysis suggests that fibroblast-like synovial cells produced c19orf10, whereas macrophages, B cells, or T cells produced little or none of this protein. There is evidence of secretion into the vascular space and the extracellular matrix surrounding the synovial lining. A competitive enzyme-linked immunosorbent assay confirmed the presence of microgram levels of c19orf10 in the synovial fluids of patients with one of various arthropathies. Collectively, these results suggest that c19orf10 is an FLS-derived protein that is secreted into the synovial fluid. However, the significance of this protein in synovial biology remains to be determined. The absence of known structural motifs or domains and its relatively late evolutionary appearance raise interesting questions about its function.

## Introduction

The healthy synovial membrane consists of a thin layer of fibroblast-like synoviocytes (FLSs) and macrophages. These cells produce glycosaminoglycans such as hyaluronic acid and lubricating glycoproteins for secretion into the synovial fluid [1]. Healthy homeostasis within the joint can be disturbed by development of inflammatory diseases such as rheumatoid arthritis (RA). In this situation, the synovium becomes enlarged and the cellular composition changes. The intimal layer exhibits an increased number of FLSs, there is an increase in the number of blood vessels, and the sub-intima becomes infiltrated with lymphocytes and plasma cells forming ectopic lymphoid follicles [2]. The phenotype of the synovial cells also changes. *In vitro*, RA FLSs exhibit a transformed phenotype reminiscent of that seen in tumors (increased proliferative potential and resistance to apoptosis), whereas the vascular endothelium displays an increased ratio of apoptotic to proliferative cells, which is indicative of vascular remodeling [3]. These changes may contribute to the maintenance of synovial inflammation, aggravating the destruction of cartilage and bone and stimulating the development of the pannus. Because FLSs can exhibit significant phenotypic changes under different pathological conditions, studies were initiated to examine the repertoire of proteins produced by these cells. Ultimately, these studies could provide information about differences in protein synthesis by FLSs in health and disease.

Previously, proteomic studies were initiated to determine the protein composition and expression patterns of FLSs [4]. One of the proteins identified was a major FLS protein encoded by the novel gene, c19orf10 (chromosome 19 open reading frame 10). *c19orf10 *has been identified in two other reports in the literature. Tulin and colleagues [5] performed a genetic complementation screening approach looking for stromal cell-derived factors involved in cell proliferation and identified c19orf10 as a murine bone marrow stroma-derived growth factor (SF20/interleukin [IL]-25). Subsequent work revealed that the proliferation data described in the initial report could not be replicated and the report was withdrawn [6]. In another report, Wang and colleagues [7] profiled the proteins secreted from pre-adipocytes (3T3-L1) by means of two-dimensional electrophoresis (2DE) mass spectrometry (MS). The authors identified a number of secreted proteins, including c19orf10 (SF20/IL-25), which was upregulated during differentiation of adipocytes, leading to the suggestion that it is involved in adipogenesis. Although these studies have postulated that c19orf10 is involved in cell proliferation and differentiation, none of these studies has revealed any functional information about the molecule. We undertook the characterization of c19orf10 in the synovium because it is a quantitatively significant product of FLSs [4] and little is known about the processing and function of the c19orf10 molecule.

## Materials and methods

### Cells, tissues, and synovial fluid

Synovial tissue was obtained with informed consent from patients with RA or osteoarthritis (OA) at the time of knee or hip arthroplasty. All patients with RA met American College of Rheumatology criteria [8]. FLSs were isolated and cultured as previously described [9]. Synovial fluid was obtained with informed consent from five patients diagnosed with RA, reactive arthritis, or gout. All samples were obtained according to the guidelines approved by the Ethics Committee of the University of Manitoba (Winnipeg, MB, Canada).

### Sample preparation, two-dimensional electrophoresis analysis, in-gel digestion, and mass spectrometry

Cell lysates were prepared and isolated as previously described [4]. The preparative 2DE and MS were performed as described by Dasuri and colleagues [4]. Digests were analyzed using a matrix-assisted laser desorption/ionization quadrupole time-of-flight mass spectrometer [10], and proteins were identified by single MS (peptide mass fingerprinting) using ProFound [11] and by tandem mass spectrometry (MS/MS) using Tandem search engine [12,13]. The National Center for Biotechnology Information (NCBI) (Bethesda, MD, USA) non-redundant human database was used in both cases.

### cDNA cloning and expression constructs

A full-length c19orf10 IMAGE (Integrated Molecular Analysis of Genomes and their Expression) clone (4562455) corresponding to GenBank accession number NM_019107 was obtained from Open Biosystems (Huntsville, AL, USA). A set of oligonucleotide primers was designed to amplify the coding region of the c19orf10 sequence (amino acids 32V to 173L), excluding a putative N-terminal signal sequence. Primers Orf10F (GGTGTCCGAGCCCACGACGGT) and Orf10R1 (catggctcgaGTCACAGCTCAGTGCG) were used to amplify a 431-base pair (bp) sequence of c19orf10 encompassing nucleotides 162 to 592, excluding the region coding for the putative N-terminal signal peptide but including the 3' stop codon. This sequence was inserted into the *PshA*1 and *Xho*I sites of the pET41b vector (Novagen, part of EMD Biosciences, Inc., San Diego, CA, USA), resulting in a glutathione S transferase (GST)-His-c19orf10 fusion gene. Primers Orf10*Nde*I (gaattccatatGGTGTCCGAGCCCACGA) and Orf10R2 (catggctcgagcAGCTCAGTGCGCGAT) were used to amplify a 426-bp sequence of c19orf10 encompassing nucleotides 162 to 587, excluding both the region coding for the putative N-terminal signal peptide and the 3' stop codon. This sequence was inserted into the *Nde*I and *Xho*I sites of the pET41b vector (Novagen), resulting in a c19orf10-His fusion gene. Primers Orf10*BamH*I (catgcggatccCGGTGTCCGAGCCCA) and Orf10R1 (catggctcgaGTCACAGCTCAGTGCG) were used to amplify a 431-bp sequence of c19orf10 encompassing amino acids 32V to 173L, including the C-terminal stop codon. This sequence was inserted into the *BamH*I and *Xho*I sites of the pGEX-5X-2 vector (Amersham Biosciences, now part of GE Healthcare, Little Chalfont, Buckinghamshire, UK), resulting in a GST-c19orf10 fusion gene. Restriction enzyme analysis and DNA sequencing were used to confirm the fidelities of the plasmids. The expression constructs were then transformed into *Escherichia coli *strain BL21 or the Rosetta2 (DE3) strain enhanced with seven human tRNA genes (Novagen).

Recombinant protein expression was induced with 1 mM isopropyl-β-D-thiogalactopyranoside (Novagen) for 3 hours at 37°C. The cells were subsequently collected and frozen overnight. The frozen cell pellet was resuspended in BugBuster reagent (Novagen) in 1X phosphate-buffered saline (PBS), and the cells were lysed by treatment with lysozyme (Sigma-Aldrich, St. Louis, MO, USA) and benzonase (Novagen) for 30 minutes at room temperature and then centrifuged at 15,000*g *for 20 minutes. The supernatant was collected and applied to a Sepharose nickel affinity column (Novagen) or a Sepharose GST affinity column (Novagen) according to the manufacturer's instructions. Rhc19orf10-His tag was eluted from the Sepharose nickel affinity column with 300 mM imidazole. Recombinant GST-tagged c19orf10 was eluted from the glutathione column with 10 mM reduced glutathione. Unlabelled c19orf10 (amino acids 32 to 173) was produced by digestion of GST-c19orf10 with Factor Xa for 4 hours at room temperature and then purified through another GST affinity column. The released recombinant protein was collected in the effluent. Protein quality and purity were assessed by 12% SDS-PAGE and visualized with Imperial purple protein stain (Pierce, Rockford, IL, USA).

### Antibody production

Monoclonal antibodies were generated in mice as previously described [14]. Female BALB/c mice were immunized with recombinant c19orf10 (rhc19orf10) fusion protein containing both GST and s (GST-His-c19orf10) or c19orf10-His. Ten or twenty-five micrograms of c19orf10 fusion protein was mixed with Titer-Max Gold adjuvant (Cedarlane Laboratories Ltd., Burlington, ON, Canada) and administered subcutaneously. The mice were boosted 1 month later. Three months later, one mouse was boosted without adjuvant intraperitoneally 4 days prior to the fusion. After the fusion, hybridomas that were enzyme-linked immunosorbent assay (ELISA)-positive to c19orf10 were selected for further analysis and cloning. Cloned hybridomas were grown to death in RPMI-1640 containing 10% fetal bovine serum, and supernatants were collected and used as a source of antibodies. The antibodies were characterized using an Isotyping Monoclonal Antibodies Kit from GE Healthcare. One of the anti-rhc19orf10 clones, 1B6, was grown in serum-free media, and antibody was purified using a KaptivM column (BioCan Scientific, now part of MediCorp Inc., Montreal, QC, Canada).

The ELISA studies were performed using Nunc Maxisorp multi-well plates (Nalge Nunc, Naperville, IL, USA) coated overnight at 4°C with 1.0 μg/well of c19orf10-His fusion protein. Plates were washed and then blocked with 1% bovine serum albumin (BSA) in PBS. The plates were incubated either with primary antibodies in culture supernatants containing 10% serum or with purified antibody. The plates were incubated with goat anti-mouse immunoglobulin (Ig) G (whole molecule) alkaline phosphatase conjugate (Sigma-Aldrich), and the reaction was developed using *p*-nitrophenyl phosphate substrate (Sigma-Aldrich). The response was quantified at 405 nm on an ELISA plate reader.

A chicken polyclonal antibody was produced by immunization with recombinant His-tagged c19orf10 (Gallus Immunotech, Inc., Fergus, ON, Canada). A hen was immunized twice with 100 μg of orf10-His and then twice with 50 μg of orf10-His. Immune eggs were collected and egg yolk IgY was purified by Gallus Immunotech, Inc. A competitive ELISA was developed using this antibody. Nunc Maxisorp multi-well plates were coated overnight at 4°C with GST-c19orf10 fusion protein (0.8 μg/well). Plates were washed and then blocked with 1% BSA in PBS. The plates were incubated with anti-c19orf10 IgY or anti-c19orf10 IgY pre-incubated with GST-c19orf10 or synovial fluid. The plates were washed and incubated with donkey anti-chicken IgY horseradish peroxidase (HRP) conjugate (1:15,000) (Gallus Immunotech, Inc.). The reaction was monitored with tetramethylbenzidine ELISA substrate solution (Sigma-Aldrich) and read at 450 nm.

### Immunofluorescence

FLSs were stained with monoclonal anti-rhc19orf10 antibodies. Twenty-one-spot microscope slides (Erie Scientific Company, Portsmouth, NH, USA) coated with 5 or 10 μg/ml of fibronectin (Sigma-Aldrich) were seeded with 5 × 10^4 ^cells per spot and incubated overnight in 37°C with 10% CO_2_. After incubation, the cells were washed in PBS and then fixed in 4% paraformaldehyde (Polysciences, Inc., Warrington, PA, USA) in PBS for 15 minutes. The cells were then washed and, in some cases, permeabilized using 0.2% Triton X-100 (Sigma-Aldrich) in PBS for 5 minutes and washed again. The slides were treated with supernatants containing anti-rhc19orf10 antibodies or purified antibody for 1 hour at room temperature. Excess primary antibody was removed with three washes of PBS, and the cells were then treated with Oregon Green 488-conjugated phalloidin (Invitrogen Corporation, Carlsbad, CA, USA) in combination with cyanine 3-conjugated goat anti-mouse Ig (Jackson ImmunoResearch Laboratories, Inc., West Grove, PA, USA). Fluorescence was visualized using an epifluorescence microscope (Olympus BX60; Olympus, Tokyo, Japan) equipped with a xenon arc lamp, light pipe (Lambda LS; Sutter Instrument Company, Novato, CA, USA), and a Sensicam digital camera (The Cooke Corporation, Romulus, MI, USA). Images were processed with Image-Pro software (Media Cybernetics, Inc., Silver Spring, MD, USA).

### Immmunohistochemistry

Fresh frozen synovial biopsies were sectioned at 5 μm using a cryostat. Two sequential sections were placed side-by-side on a charged microscope slide (ProbOn; Fisher Scientific Co., Pittsburgh, PA, USA). Tissue staining was carried out using Dako's ABC system (Dako North America, Inc., Carpinteria, CA, USA). Tissue sections were fixed in chilled acetone, air-dried, and rehydrated in PBS. Endogenous tissue peroxidase was blocked by incubating the sections with hydrogen peroxide solution (Dako North America, Inc.). Sections were also blocked by incubation with normal serum from the animal species used for secondary antibody generation. Primary antibodies were then added to each tissue section and incubated overnight at 4°C in a humidified slide chamber. The appropriate biotinylated secondary antibody, streptavidin-HRP, and diaminobenzidine substrate (all from Dako North America, Inc.) were used to detect the binding of the primary antibodies. Murine Ig of irrelevant specificity was added to a tissue section adjacent to the primary antibody and used as a negative control.

Paraffin blocks were prepared by immersing tissue samples in neutral buffered 10% formalin for 8 hours for fixation. They were dehydrated in ascending graded ethanol and infiltrated and embedded in low-melting paraffin at 56°C in a heated oven. The tissue-paraffin mold was solidified on a cold plate to form a block. Four-micrometer sections were cut by a microtome. The slides were then rehydrated in descending graded ethanol and processed through the same immunostaining steps as for frozen sections.

## Results

**Figure 1:**
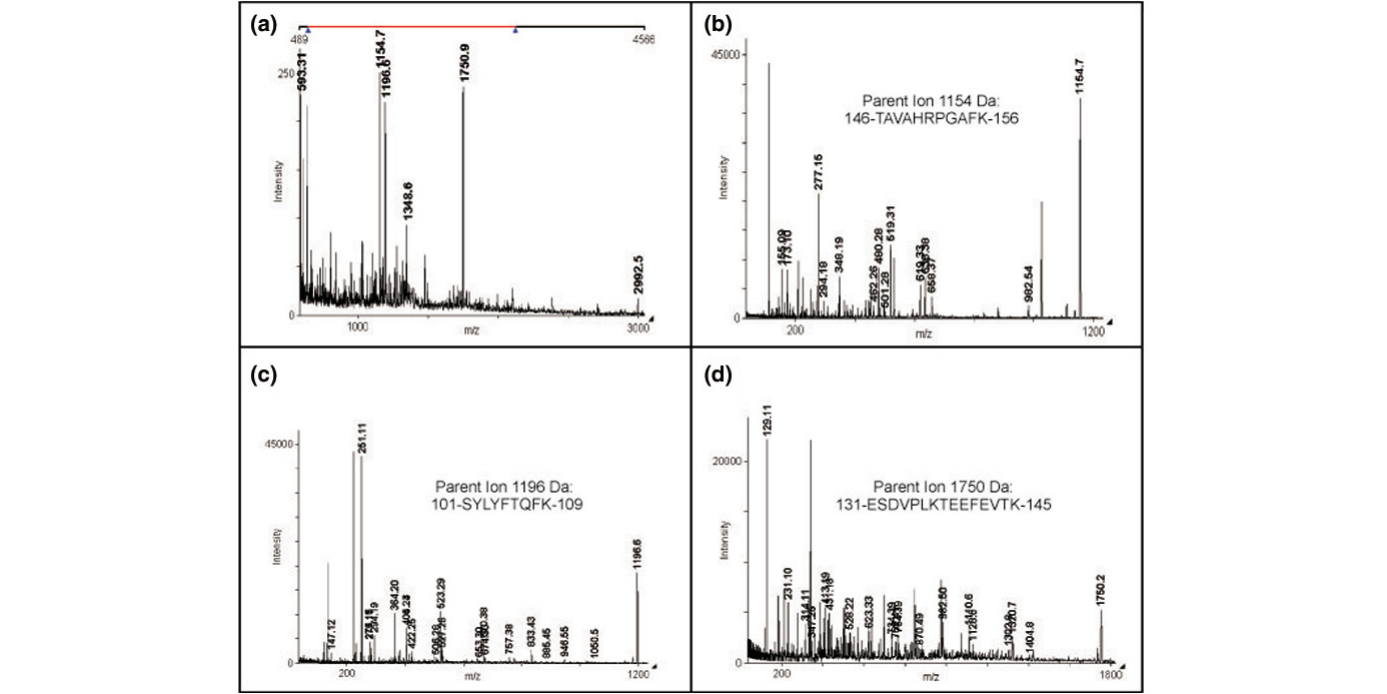
Proteomic identification of c19orf10. **(a) **Mass spectra of in-gel digest of c19orf10 with six peptides mapping to the c19orf10 protein labeled. **(b) **Tandem mass spectrum of c19orf10 parent ion 1,154 Da, 146-TAVAHRPGAFK-156. **(c) **Tandem mass spectrum of c19orf10 parent ion 1,196 Da, 101-SYLYFTQFK-109. **(d) **Tandem mass spectrum of c19orf10 parent ion 1,750 Da, 131-ESDVPLKTEEFEVTK-145. Peaks contributing to the score are labeled. The mass and sequence of each parent ion are indicated on the appropriate spectrum. c19orf10, chromosome 19 open reading frame 10.

Mass spectrometric analysis of two-dimensional SDS-PAGE-separated synovial fibroblast lysates identified a 16.5-kDa spot as the product of c19orf10 [4]. Total peptide coverage corresponded to 47% of the predicted peptide sequence with a ProFound expectation score of 2.5 × 10^-6 ^[11] (Figure 1a). Subsequent MS/MS analysis of three of the peptides derived from this spot confirmed that the sequence corresponded to that of c19orf10 with a GPM log (e) score of -16.8 (Figure 1b–d) [12]. Furthermore, manual MS/MS analysis of a 2,992.5-Da parent ion indicated that it was a non-tryptic peptide corresponding to amino acids 32 to 60.

**Figure 2:**
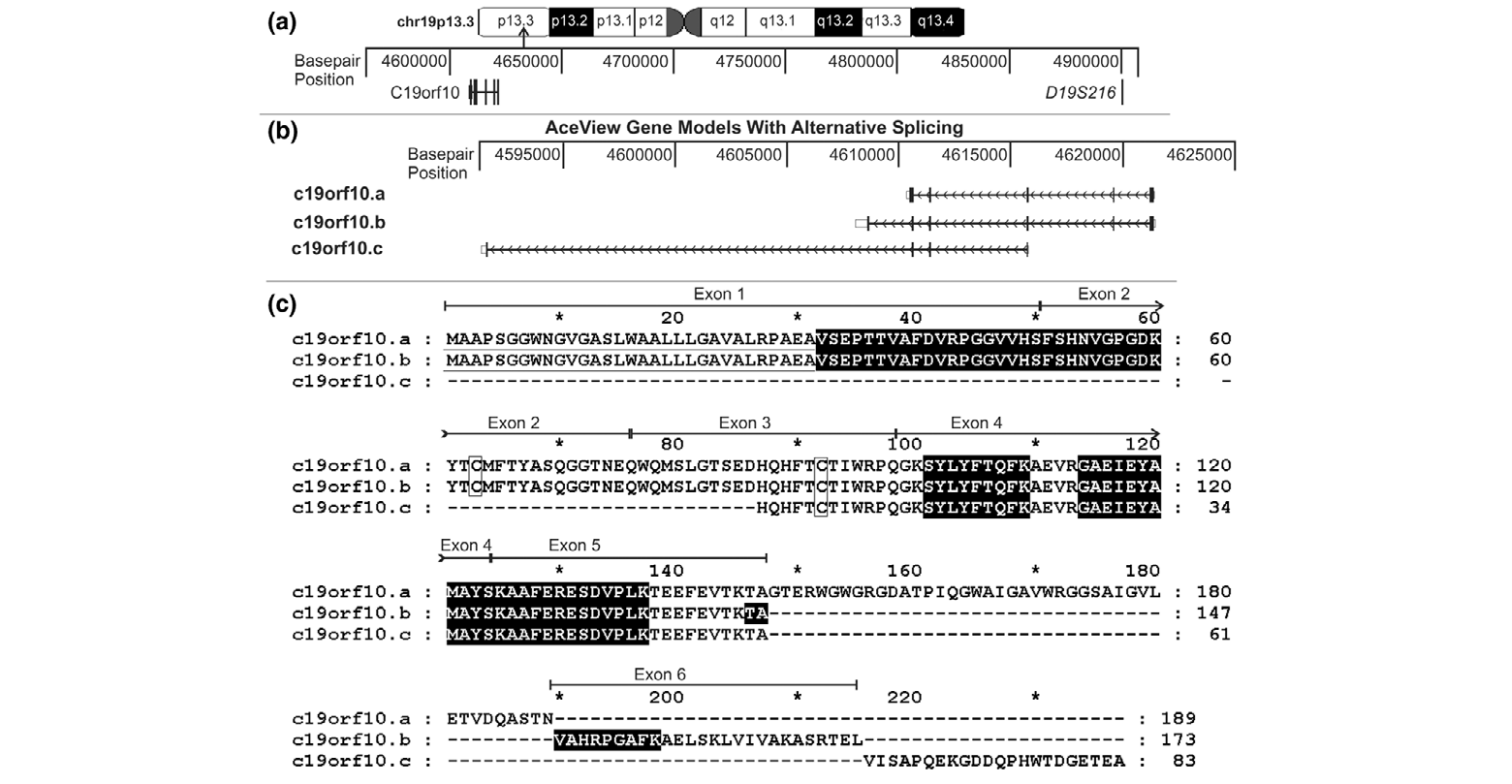
Genomic organization, alternative splicing, and protein sequence of c19orf10. **(a) **The chromosomal localization of the region containing the c19orf10 gene is indicated on the ideogram. The area containing the c19orf10 gene is expanded and the base-pair positions are indicated. The location of a microsatellite marker linked to juvenile rheumatoid arthritis, *D19S216*, is also indicated. **(b) **The expanded region of chromosome 19 containing the c19orf10 gene. Three putative splicing variants are indicated (c19orf10.a, c19orf10.b, and c19orf10.c). Thick blocks indicate translated exons, open blocks indicate untranslated exons, horizontal lines indicate introns, and the arrows indicate the direction of transcription. **(c) **Alignment of protein products of c19orf10 splicing variants. Variants a and b seem to be complete sequences starting with an N-terminal methionine. Variant c does not start with an N-terminal methionine and is probably incomplete at the N-terminus. Lines above the sequence map the exons to the protein sequence. Shaded sequences indicate peptides observed by mass spectrometry. The putative N-terminal signal peptide is underlined with a solid black line. C63 and 92 are indicated by rectangles. c19orf10, chromosome 19 open reading frame 10.

The c19orf10 gene is located on chromosome 19p13.3 and spans approximately 30 kbp [15] (Figure 2). A survey of the available cDNA clones suggests the possibility of three splice variants for c19orf10 [16] (Figure 2b). The most common variant, c19orf10.b, has six exons and is supported by sequence data from 468 clones. The other two predicted variants (a and c) have been identified with sequence data support from one clone each. It is likely that variant c is incomplete at the 5' end. Predicted protein products of the three splice variants are illustrated in Figure 2c. All of the c19orf10 peptides identified by our analysis are present in the c19orf10.b sequence (highlighted peptides, Figure 2c). However, based on the predicted molecular weight of variant c, it is not expected to be in the same molecular weight regions on the gels as the variants a and b. Hence, it is not possible to comment on its presence in the FLSs.

The c19orf10 gene product variant c is predicted to be a 173-amino acid protein with a theoretical molecular weight of 18.8 kDa and a theoretical isoelectric point of 6.2. Two cysteine residues, at positions 63 and 92 (Figure 2c, outlined with a rectangle), are predicted to be disulfide-bonded [17]. A 31-amino acid signal peptide is predicted using the SignalP [18] (and PSORT II [19] algorithms (Figure 2c, underlined), suggesting that this is a secreted protein. The presence of a cleavable signal peptide is consistent with the results of the MS/MS analysis, which found a non-tryptic cleavage site at position 32. This corresponds to the exact site at which the signal peptide is predicted to be cut to generate the mature protein.

The c19orf10 sequence was also examined for other sequence patterns that might predict molecular features or properties. Domain and pattern searches using InterProScan to query ProDom, PFAM, SMART, and PRINTS [20] suggest that c19orf10 does not possess any known domains or motifs. Two potential O-glycosylation sites are predicted at threonines 36 and 37 using NetOGlyc3.1[21]. The fact that the 2,992.5-Da peak corresponding to the N-terminal peptide containing unmodified threonines 36 and 37 was observed suggests that not all of the protein is necessarily glycosylated. Eight potential phosphorylation sites at residues S33, S84, S132, S169, T36, Y61, Y67, and Y119 are predicted by the NetPhos 2.0 server [22]. These results suggest that c19orf10 is a secreted protein with unique structural features.

A panel of murine monoclonal antibodies was produced against rhc19orf10 for immunohistochemistry and immunoassays in an effort to define the distribution of c19orf10 in synovium. Three hybridomas were selected based on reactivity with rhc19orf10. The antibodies are very effective in immunofluorescence as well as immunohistochemistry on both frozen and paraffin sections, but they do not work on Western blot. All of the antibodies give similar patterns of reactivity in a comparative staining analysis. These antibodies were all of the IgM class and this may be attributable to the fact that there is 91% identity between the predicted mature human and murine proteins. Thus, the immunogenicity of c19orf10 would be expected to be low and an isotype switch to IgG would be more difficult to achieve.

**Figure 3:**
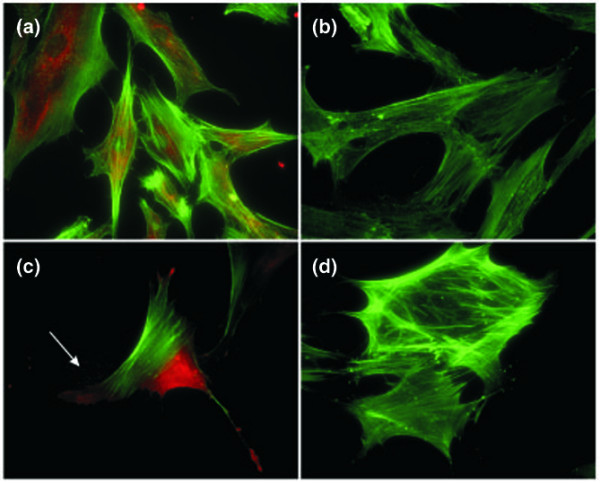
c19orf10 immunofluorescence staining of fibroblast-like synoviocytes (FLSs). **(a) **FLSs were labeled with anti-c19orf10 monoclonal antibody, 1B6, and visualized using red fluorescent cyanine 3 (Cy3) goat anti-mouse immunoglobulin G (IgG) (heavy and light chain reactive [H&L]) antibody. The cells were counterstained with green fluorescent Oregon Green phalloidin to visualize the F-actin. **(b) **Negative control with no primary antibody, stained as above. **(c) **FLSs were labeled with anti-c19orf10 monoclonal antibody, 1B6, and visualized using red fluorescent Cy3 goat anti-mouse IgG (H&L) antibody. The cells were counterstained with green fluorescent Oregon Green phalloidin to visualize the F-actin (arrow). **(d) **Negative control with no primary antibody, stained as above. c19orf10, chromosome 19 open reading frame 10.

The staining of permeabilized low-passage FLSs with the monoclonal antibody, 1B6, revealed a perinuclear punctate distribution, which was consistent with an endoplasmic reticulum/Golgi distribution (Figure 3a,c). This staining pattern was detected with the other antibodies to c19orf10, whereas there was no evidence of staining with control antibodies (Figure 3b,d). In many (but not all) cases, there appeared to be a faint staining on the substrate surrounding the cells (Figure 3c, arrow), suggesting that the protein was secreted.

**Figure 4:**
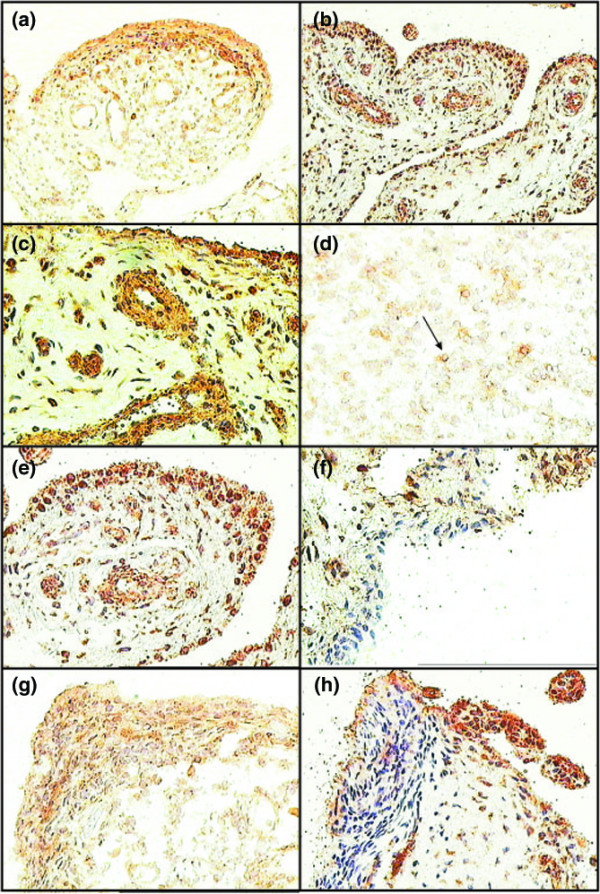
Expression of c19orf10 in rheumatoid arthritis (RA) and osteoarthritis (OA) synovium. **(a,d,f,h) **Expression of c19orf10 in RA synovium. **(b,c,e,g) **Expression of c19orf10 in OA synovium. **(a) **Intense staining of the synovial lining layer and perivascular regions of RA (OCT section) tissue.**(b) **Intense staining of the synovial lining layer and perivascular regions of OA (paraffin section) tissue. **(c) **An area demonstrating a thin lining layer and perivascular region populated with c19orf10-positive cells. Note that the sublining stroma in this area is virtually devoid of c19orf10 staining. This pattern of staining is typical of that seen in both RA and OA sections. **(d) **In most lymphocytic aggregates, there was minimal staining of the lymphocytes although some mononuclear cells in the aggregates stained positively (arrow). **(e) **Intense staining of individual cells in the lining layer of a typical OA synovium. **(f) **An area of an OA synovium demonstrates a lining layer completely devoid of c19orf10 staining. **(g) **Intense staining of a hyperplastic RA synovial lining cell layer. This staining was typical of most areas of RA synovium where the lining was hyperplastic. **(h) **An area of an RA synovial lining layer that is not positive for c19orf10 staining. c19orf10, chromosome 19 open reading frame 10.

The distributions of c19orf10 in the synovial tissues from patients with RA or OA are very similar (Figure 4). The majority of staining is in the synovial lining with some cells interspersed in the deeper tissues (Figure 4a,b). Staining in the perivascular regions of the small vessels is also noted (Figure 4c). In these cases, the staining appears to be largely extracellular, suggesting that the secreted proteins are bound at these sites. In regions of high mononuclear cellularity (Figure 4d), there is limited intermittent staining of a subset of cells. In addition, areas of synovial hyperplasia show variable staining patterns. In some cases, areas of hyperplasia are positive for c19orf10 staining (Figure 4e,g), whereas in other cases, there is an apparent absence of c19orf10 staining (Figure 4f,h). These observations raise the possibility that there may be either functional or compositional changes in the synovial cellular content which result in altered synthesis of this protein. Clearly, the significance and relationship to disease will require detailed comparative analysis.

**Figure 5:**
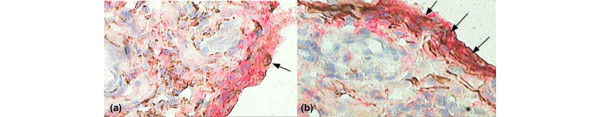
The cellular origins of c19orf10 in the synovium. Sections of rheumatoid arthritis synovial tissue stained with anti-c19orf10 stained red. **(a) **Sections stained with anti-CD68 to detect macrophage-like cells stained brown (arrow). **(b) **Sections stained with anti-CD55 to detect fibroblasts stained brown (arrows). There was a clear colocalization with CD55-staining cells, whereas the association with CD63-positive cells was much less apparent. c19orf10, chromosome 19 open reading frame 10.

The cellular origins of the synovial tissues were examined using double-staining for c19orf10 and either CD68 as a marker for macrophage-like cells or CD59 as a marker for fibroblasts. There is clear colocalization of CD59 and c19orf10 staining throughout the synovial lining and in the underlying tissues (Figure 5). This contrasts with the situation with CD68, in which there was no obvious association between the bulk of c19orf10 distribution and the presence of CD68, suggesting that these cells are not the major producers of c19orf10 in the tissues examined. Similarly, staining for B cells (CD20) and T cells (CD25) also failed to show any evidence of codistribution with c19orf10 (data not shown).

**Figure 6:**
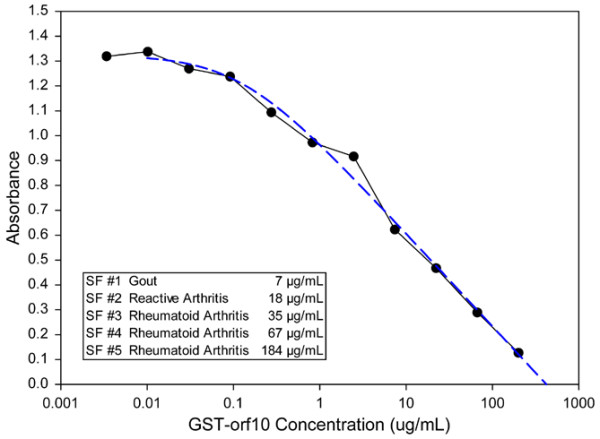
Demonstration of c19orf10 in synovial fluids. Synovial fluid c19orf10 levels were determined by competitive enzyme-linked immunosorbent assay for five patients with the indicated arthropathies. Each fluid was measured at two different dilutions. The concentrations for each are indicated in the table. A representative standard curve is presented in the graph. c19orf10, chromosome 19 open reading frame 10; GST-orf10, glutathione S transferase-open reading frame 10; SF, synovial fluid.

In some tissue sections, c19orf10 deposition was observed on the luminal surface of the synovium, suggesting that it was being secreted into the joint space. We developed a competitive inhibition ELISA assay as a direct test of this possibility to measure the c19orf10 levels in synovial fluid. Initial assays using the murine IgM hybridomas were unsatisfactory for this purpose, so chicken polyclonal antibodies were produced. Chicken is phylogenetically more distant from humans than mouse is, and as such there is greater divergence in the c19orf10 sequence (see below). This was expected to increase the immunogenicity of the human c19orf10. A hen was immunized with c19orf10-His fusion protein and the reactivity and specificity of the purified IgY were confirmed using GST-c19orf10 and a panel of unrelated His and GST fusion proteins. The antibody was then used to establish a competitive ELISA for the detection of c19orf10 in synovial fluids (Figure 6). The synovial fluids from five patients with different arthropathies were examined for the presence of c19orf10. The c19orf10 levels ranged from 7 to 184 μg/ml. This corresponds to millimolar concentrations of the protein. Specificity controls confirmed that only c19orf10 was detected in this assay, lending further support to the contention that this protein represents a major component of synovial fluid. At this time, it is unwarranted to try to draw any links between the relative fluid levels of c19orf10 and the various diseases. The only clear conclusion that can be drawn is that c19orf10 is present in synovial fluids.

The tissue distribution of c19orf10 is unknown at this time; however, based on the reverse transcription-polymerase chain reaction studies of Tulin and colleagues [5], it appears that the protein may be broadly expressed. High levels of gene expression were observed in testis, spleen, and heart, and moderate levels were observed in the lung and liver. In contrast, brain, kidney, and skeletal muscle did not show any expression. Significant expression levels were found in carcinomas of the lung, breast, and colon, whereas respective healthy tissues were negative. Resting CD8^+ ^cells, CD19^+ ^cells, and mononuclear cells were shown to have significant c19orf10 expression. Conversely, resting CD4^+ ^and CD14^+ ^cells were negative for c19orf10 as were activated CD19^+ ^cells, CD4+ cells, and mononuclear cells. The proteomic studies of Wang and colleagues [7] identified c19orf10 as a secreted product of 3T3 fibroblasts as they differentiate into adipocytes. Collectively, these observations suggest that c19orf10 may not be unique to the synovial compartment; however, the results of the present study clearly indicate that it is a significant synovial fluid component, at least in some disease states.

**Figure 7:**
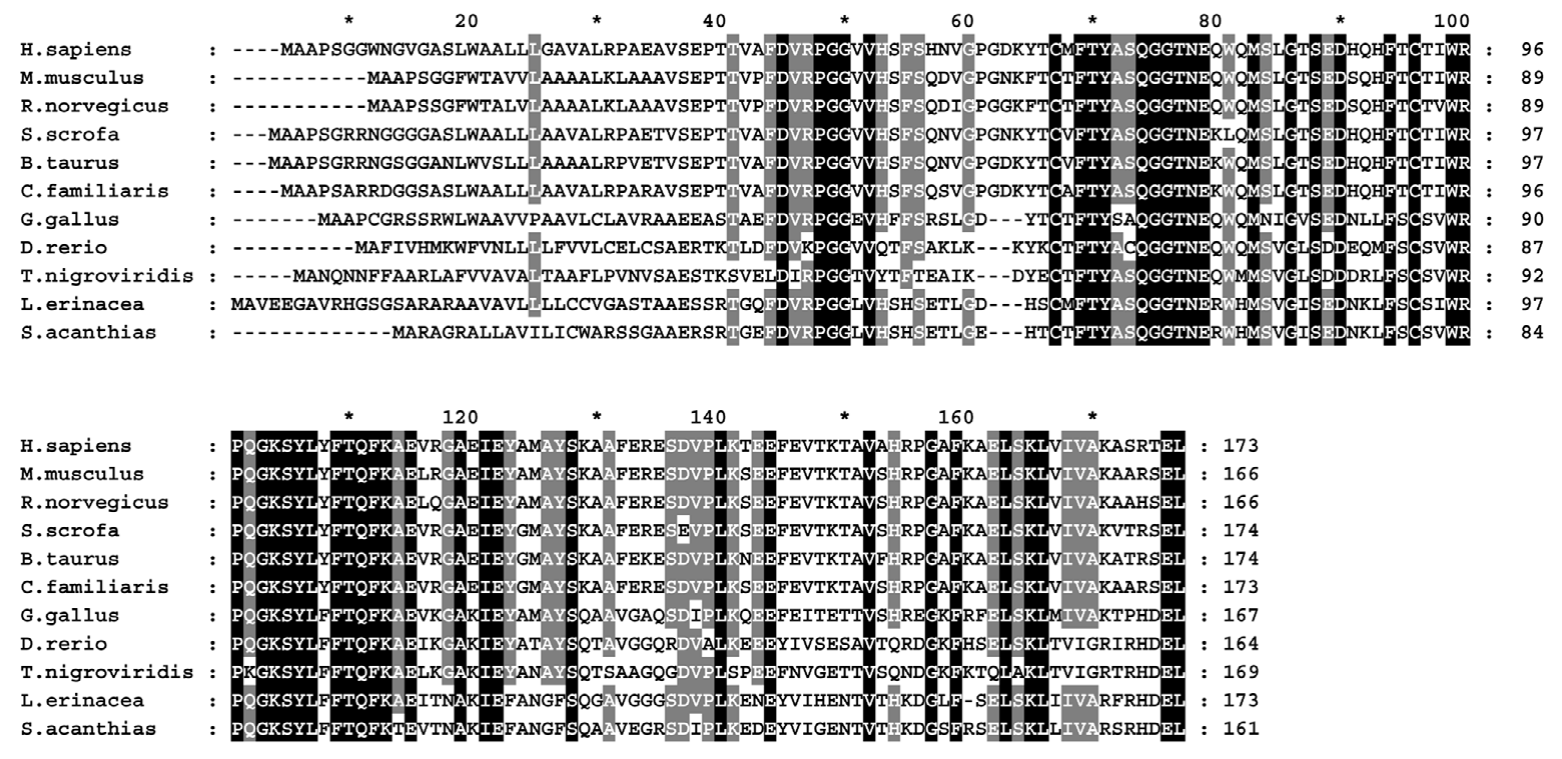
The presence of c19orf10 homologues is predicted in multiple vertebrate species. All sequences were obtained from GenBank [25]. *Homo sapiens*, NP_061980; *Mus musculus*, NP_543027; *Rattus norvegicus*, XM_347126; *Sus scrofa*, SSC.4092; *Bos taurus*, NP_01001164; *Canis familiaris*, ENSCAFT30192; *Gallus gallus*, NP_001006342; *Danio rerio*, NP_001002480; *Tetraodon nigroviridis*, CAG08012; and *Leucoraja erinacea*, CV067465 (translated in reading frame +3); *Squalus acanthias*, CX789984 (translated in reading frame +2). c19orf10, chromosome 19 open reading frame 10.

Extensive database searches identified c19orf10 homologues in 18 species of vertebrates, including mammals, amphibians, birds, and fishes. The c19orf10 aliases identified in the databases include R33729 (EMBL|CAB96948.1) and lymphocyte antigen 6 complex, locus E ligand (ly6e, GB|NP_543027.1). Several of the sequences are not full-length and were omitted from further analyses. A multiple alignment of the remaining sequences was performed using the T-Coffee server [23] (Figure 7). The sequences were similar in size, ranging from 161 to 174 amino acids. The N-terminal 40 residues in the area of the putative signal peptide were poorly conserved although the majority of the residues were non-polar. The remainder of the protein showed a high degree of conservation between the human sequence and all other sequences (Figure 7). The two cysteine residues at positions 63 and 92 of the human sequence were completely conserved, consistent with the prediction that they participate in a disulfide bond. Twenty-eight percent of the residues are identical across the species listed, and an additional 19% of the residues are highly conserved.

We have chosen not to assign a name to the *c19orf10 *product at this time because there is no clear understanding of the function of this protein. The previous name, IL-25, was based on an apparent growth-factor activity [6]. The claim of this activity was subsequently retracted and this has led to significant confusion in the literature and the databases regarding the designation IL-25. c19orf10 does not show any similarity to either IL-25 or IL-27. The IL-25 annotation has been changed in the NCBI databases, and the HUGO (Human Genome Organisation) Gene Nomenclature Committee (London, UK) [24] has indicated that the designations IL-25 and IL-27 would not be used. We suggest that *c19orf10 *not be named at this time.

## Conclusion

The protein encoded for by *c19orf10 *displays novel structural features. The protein is produced by cultured fibroblast-like synovial cells and cells with fibroblast markers in synovial tissues. The *c19orf10 *protein is found in significant concentrations in the synovial fluids of patients with one of a variety of arthropathies. The patterns of synthesis in the synovium may change with synovial tissue cellularity, possibly indicating that alterations in production rates or biosynthetic capabilities may occur in subsets of synovial fibroblasts in RA and OA. However, the role of this protein in synovial biology in both health and disease remains to be determined.

## Abbreviations

2DE = two-dimensional electrophoresis; bp = base pair; BSA = bovine serum albumin; c19orf10 = chromosome 19 open reading frame 10; ELISA = enzyme-linked immunosorbent assay; FLS = fibroblast-like synoviocyte; GPM = Global Proteome Machine; GST = glutathione S transferase; HRP = horseradish peroxidase; Ig = immunoglobulin; IL = interleukin; MS = mass spectrometry; MS/MS = tandem mass spectrometry; NCBI = National Center for Biotechnology Information; OA = osteoarthritis; PBS = phosphate-buffered saline; RA = rheumatoid arthritis; rhc19orf10 = recombinant chromosome 19 open reading frame 10.

## Competing interests

JAW, HE-G, and OK hold a provisional patent, 'CHROMOSOME19 ORF10: Molecular properties and distribution' (US Patent and Trademark Office serial number 60/746,828). The remaining authors declare that they have no competing interests.

## Authors' contributions

TW participated in the design and analysis of the study, drafted the manuscript, performed c19orf10 *in silico *analysis and the multiple sequence alignment, purified recombinant protein, and carried out the immunofluorescence, immunohistochemistry, and ELISA assays. QD cloned c19orf10 constructs and generated recombinant protein. OK analyzed the MS data. WE and KS provided instrumentation. HE-G performed immunohistology and reviewed the manuscript. JAW initiated the study, planned the studies, and revised the manuscript. All authors read and approved the final manuscript.
